# Relationship between the Expression of CHK2 and p53 in Tumor Tissue and the Course of Papillary Thyroid Cancer in Patients with *CHEK2* Germline Mutations

**DOI:** 10.3390/cancers16040815

**Published:** 2024-02-17

**Authors:** Danuta Gąsior-Perczak, Artur Kowalik, Janusz Kopczyński, Paweł Macek, Kornelia Niemyska, Agnieszka Walczyk, Krzysztof Gruszczyński, Monika Siołek, Tomasz Dróżdż, Marcin Kosowski, Iwona Pałyga, Piotr Przybycień, Olga Wabik, Stanisław Góźdź, Aldona Kowalska

**Affiliations:** 1 Collegium Medicum, Jan Kochanowski University, 25-317 Kielce, Poland; pawel.macek@onkol.kielce.pl (P.M.); a.walczyk@post.pl (A.W.); tomasz.drozdz@ujk.edu.pl (T.D.); markos86@vp.pl (M.K.); iwonapa@tlen.pl (I.P.); stanislawgo@onkol.kielce.pl (S.G.); aldonako@onkol.kielce.pl (A.K.); 2 Endocrinology Clinic, Holycross Cancer Centre, S. Artwińskiego St. 3, 25-734 Kielce, Poland; przybycien.piotr@o2.pl; 3 Department of Molecular Diagnostics, Holycross Cancer Centre, S. Artwińskiego Str. 3, 25-734 Kielce, Poland; artur.kowalik@onkol.kielce.pl (A.K.); gruszczynski.k@wp.pl (K.G.); 4 Division of Medical Biology, Institute of Biology, Jan Kochanowski University, Uniwersytecka 7, 25-406 Kielce, Poland; 5 Surgical Pathology, Holycross Cancer Centre, S. Artwińskiego Str. 3, 25-734 Kielce, Poland; januszko@onkol.kielce.pl (J.K.); kornelia.niemyska@wp.pl (K.N.);; 6 Department of Epidemiology and Cancer Control, Holycross Cancer Center S. Artwińskiego St. 3, 25-734 Kielce, Poland; 7 Genetic Clinic, Holycross Cancer Centre, 25-734 Kielce, Poland; monika.siolek@wp.pl; 8 Department of Radiology, Holycross Cancer Centre, S. Artwińskiego Str. 3, 25-734 Kielce, Poland; 9 Clinical Oncology, Holycross Cancer Centre, S. Artwińskiego Str. 3, 25-734 Kielce, Poland

**Keywords:** papillary thyroid cancer, *CHEK2* mutation, checkpoint kinase 2, p53, *TP53* gene, immunohistochemistry, fluorescence in situ hybridization

## Abstract

**Simple Summary:**

CHK2 has functions in DNA damage repair. Germline *CHEK2* mutations impair these functions, increasing the risk of PTC. The aim of this study was to determine whether CHK2 and p53 expression in patients with a germline *CHEK2* mutation in tumor tissue can serve as a prognostic marker for papillary thyroid cancer (PTC), and whether copy number aberrations in *CHEK2* and *TP53* correlate with the course of PTC. This study included 156 patients with PTC who had been selected from a group of 1547 people previously tested for the presence of *CHEK2* mutations in their peripheral blood. Higher CHK2 expression was associated with poorer treatment responses and disease outcomes in PTC patients. Higher CHK2 expression and positive p53 together with a TP53 deletion could have potential as a prognostic marker of unfavorable disease outcomes in patients with germline truncating mutations in *CHEK2*.

**Abstract:**

The aim of this study was to determine whether the expression of CHK2 and p53 in tumor tissue in carriers of germline *CHEK2* mutations can serve as a prognostic marker for PTC, and whether *CHEK2* and *TP53* copy numbers correlates with the course of PTC disease. This study included 156 PTC patients previously tested for the presence of *CHEK2*. Clinicopathological features, treatment response, disease outcome, and germline mutation status of the *CHEK2* gene were assessed with respect to CHK2 and p53 expression, and *CHEK2* and *TP53* gene copy statuses. In patients with and without a germline mutation in *CHEK2* and with higher CHK2 expression, the chances of an excellent treatment response and no evidence of disease were lower than in patients without or with lower CHK2 expression. *TP53* deletion was associated with angioinvasion. In patients with a truncating mutation, the chance of a *CHEK2* deletion was higher than in patients with WT *CHEK2* alone or those with WT *CHEK2* and with the missense I157T mutation. Higher CHK2 expression was associated with poorer treatment responses and disease outcomes. Higher CHK2 expression and positive p53 together with a *TP53* deletion could be a prognostic marker of unfavorable disease outcomes in patients with germline truncating mutations in *CHEK2*.

## 1. Introduction

In recent years, there has been a steady increase in the incidence of papillary thyroid cancer (PTC) worldwide, mainly due to improved diagnostic technologies and increased awareness of screening tests. Most new cases are cancers at a low clinical stage [1,2,3]. PTC currently has an excellent prognosis [4]. However, in some patients, PTC can lead to metastases and death of the patient. The mortality of patients with differentiated thyroid cancer is approximately 1–2% [5]. The molecular mechanisms determining this mortality are still largely unknown. Thus, it is extremely important to identify molecular markers not only in tumor tissue after surgery but, more importantly, in peripheral blood before surgery to select for patients in need of aggressive treatment. Mutations in DNA repair pathway genes (ATM/CHK2/p53) represent one potential source of markers. The checkpoint kinase 2 gene (*CHEK2*) is involved in DNA repair and is activated by DNA damage, especially double-strand breaks. It is also a tumor suppressor gene. It is located on the long arm of human chromosome 22 at position q12.1 and encodes a protein (CHK2, checkpoint kinase 2) with serine-threonine kinase activity. CHK2 regulates the cell cycle and prevents uncontrolled cell division. In response to a double-stranded DNA break, the serine- threonine kinase ATM (ataxia-telangiectasia mutated gene) is activated and phosphorylates and activates CHK2, which then phosphorylates proteins involved in the DNA damage response, such as p53, Cdc25A, Cdc25C, and BRCA1, which leads to cell cycle arrest in the G1, S, and G2 phases and initiation of DNA damage repair. However, if the DNA damage is irreparable, the cells undergo cell death in a process termed apoptosis. These observations suggest that CHK2 is regulated at sites of double-stranded DNA breaks and plays an important role in the DNA damage response. Therefore, an efficient signaling ATM/CHK2/p53 pathway is essential for the coordination of DNA repair, cell cycle progression, and apoptosis in response to DNA damage [6,7,8].

Mutations in the genes encoding DNA repair proteins may affect their stability and activity, which can lead to the development of cancer. Carriers of mutations in genes encoding tumor suppressor proteins, such as CHK2 and p53, are genetically predisposed to certain cancers. The expression of DNA repair pathway genes is increased in patients with malignant tumors, and this is associated with unfavorable disease outcomes in these patients. DNA repair pathway genes are also expressed at significantly higher levels in patients with melanoma metastases, suggesting that CHK2 may also mediate tumor progression [9]. A study by Zhao et al. reported that high CHK2 expression may be associated with PTC progression [10]. Increased CHK expression is also observed in most cases of diffuse large B-cell lymphoma (DLBCL), bladder cancer, and ovarian cancer, and is associated with poor clinical courses in these patients [11,12,13].

Like the *CHEK2* gene, the tumor suppressor gene *TP53* is important for maintaining genome integrity. It is referred to as the “guardian of the genome”, not alone because it is involved in the control of cell growth and division [14,15], but also because mutations in this gene commonly occur in a wide range of malignant tumors, suggesting that it suppresses cancer formation [14]. Mutations that functionally impair p53 seriously compromise the cells’ ability to repair DNA and result in apoptosis if the genetic defect is not repaired [16]. *TP53* mutations block inhibition of the G1 phase of cell cycle progression and deregulate apoptosis, resulting in the malignant transformation and proliferation of damaged cells [17,18,19]. 

Germinal *CHEK2* truncating mutations (1100delC, IVS2 + 1G > A, del5395) increase the risk of thyroid cancer by approximately 6-fold [20,21], and missense mutations such as I157T increase the risk by about 2–3 fold [20,21,22,23]. According to The Cancer Genome Atlas Research, in addition to hereditary predisposition to the development of PTC, somatic mutations occur in the *CHEK2* gene, which may contribute to PTC tumor progression [24].

We have previously shown that *CHEK2* truncating mutations, rather than missense mutations, are significantly associated with angioinvasion and an intermediate or high initial risk of recurrent/persistent PTC [21]. However, that study did not assess the expression of CHK2 and p53 in tumor tissues in carriers of *CHEK2* germline mutations with PTC.

To learn more about the role of CHK2 and p53 in PTC and determine whether they have potential as diagnostic and prognostic markers of PTC, we measured the expression of the unphosphorylated forms of CHK2 and p53 using immunohistochemistry (IHC) and determined whether their expression levels correlated with disease progression in PTC patients. Furthermore, we used fluorescence in situ hybridization (FISH) to analyze copy number aberrations of *CHEK2* and *TP53* and determine whether they correlated with disease progression in PTC patients.

## 2. Materials and Methods

### 2.1. Patients

The study included 156 PTC patients who were selected from a group of 1547 PTC patients who had previously been tested for the presence of mutations in the *CHEK2* gene in DNA isolated from peripheral blood [21]. In 43 of 51 patients from the previous study with a truncating mutation in the *CHEK2* gene, it was possible to obtain archival paraffin-embedded blocks containing fixed tissues of the primary tumor for IHC and FISH testing. Seven patients were excluded from the study due to degraded DNA. One patient with a truncating mutation in the *CHEK2* gene, who underwent thyroid surgery after 2020, from whom archival blocks were also obtained, was included in the study. A total of 44 patients with a truncating mutation were included in the study. Archival blocks containing fixed tissues of the primary tumor were obtained from 7 out of 7 patients with the I157T homozygous mutation in the *CHEK2* gene. Fifty-three of 182 patients with the I157T heterozygous mutation in the *CHEK2* gene and 52 of 1307 patients without a mutation in the *CHEK2* gene from a previous study were randomly selected according to the procedure described below.

### 2.2. Description of the Test Procedure

Patients were ordered by medical history number (in ascending order) and each patient was assigned a unique number (between 1 and 1547). Of the patients assigned to the missense mutation group, 53 were randomly selected with the use of a random number generator (R software). Of the patients assigned to the no-mutation group, 52 were randomly selected. Number 182 (the number of all patients in the missense mutation group) was selected as a setting for the random number generator in the statistical software R (open source statistical software [25]) when drawing from the group with missense mutations. The number 1307 (the number of all patients in the no-mutation group) was selected as the random number generator setting when drawing from no-mutation group in the R program.

No additional randomization was performed because it was possible to obtain archival blocks containing fixed tissues of the primary tumor from all randomly selected patients.

All study procedures were approved by the Institutional Review Board of Jan Kochanowski University, Kielce, Poland (approval number: 31/2021). All patients gave written informed consent to participate in the study. All patients were informed about the nature, objectives, and methods of the study. All patients included in the study were Caucasian. Detailed data on *CHEK2* germline mutations have been reported previously [26]. Samples of archival paraffin-embedded tumor blocks after thyroid surgery were used for immunohistochemical (IHC) analysis of *CHEK2* and p53 expression as well as for analysis of *CHEK2* and *TP53* gene copy numbers via FISH. Clinicopathological data were available for all analyzed cases and were analyzed retrospectively. All PTCs were graded histopathologically according to the 8th edition of the American Joint Committee on Cancer (AJCC) thyroid cancer staging system (AJCC/Union for International Cancer Control (UICC) TNM staging system and American Thyroid Association (ATA) modified initial risk stratification system (low, intermediate, and high risk of recurrence)) [27,28]. All patients included in the study underwent primary surgical treatment (lobectomy, total thyroidectomy, or total thyroidectomy with central or central and lateral compartment lymphadenectomy). The scope of surgery and surgical procedure have been described previously [29]. Response to therapy was assessed 1 year after initial treatment. Response to initial treatment was classified into four categories: excellent, indeterminate, biochemically incomplete, and structurally incomplete [27]. Response to treatment after thyroidectomy and I-131 radiotherapy was assessed according to Tuttle’s criteria [30], while response to treatment after thyroidectomy without I-131 radiotherapy or lobectomy was assessed according to Momesso’s criteria [31].

All procedures for monitoring the course of the disease after surgery, assessing the response to treatment, and further follow-up, as well as treatment assessment, have been described previously [32,33]. Relapse was defined as the detection of biochemical or structural disease after a period of NED (no evidence of disease at least 12 months after initial therapy) and no symptoms of the disease [27,31].

Clinical observation was completed on 30 November 2022. Patients were classified into one of the following groups based on medical records in accordance with ATA recommendations [27]: NED, indeterminate response, biochemically and structurally chronic disease, deaths from cancer, and deaths from other causes.

### 2.3. Detection of CHEK2 Mutation

DNA was isolated from whole blood samples (100 µL), collected in EDTA tubes, within 12 h of collection, using the Maxwell^®^ RSC Blood DNA Kit (Promega, Madison, WI, USA). DNA samples were eluted in 120 µL of buffer E. Genotyping and the diagnostic algorithm of molecular tests in our center have been described in detail in our previous work [21,26].

### 2.4. Fluorescence In Situ Hybridization (FISH)

*CHEK2* and *TP53* copy number determination via FISH was performed on PTC tissue fixed in 10% buffered formalin and embedded in paraffin. Four-micrometer thick paraffin sections were placed on the positively charged side of an organosilane-coated slide to minimize tissue detachment from the slide during FISH. The sections were baked for 1 h on a hot plate at 56 °C and then subjected to the protocol recommended by the probe manufacturer (CytoTest, Washington, DC, USA).

After incubation overnight, the sections were immersed twice in xylene for 10 min each and then passed through a series of decreasing alcohol concentrations (100%, 96%) for 5 min each. After rinsing twice in purified water for 5 min, the sections were immersed in pretreatment solution at 95 °C for 30 min, passed through washing buffer twice for 5 min each, and then immersed in a protease solution at 37 °C for 30 min. After washing the sections twice in washing buffer, the sections were dried at room temperature. Two probes (10 μL each) were used in parallel: the *CHEK2*/CCP22 FISH Probe (CytoTest) and the TP53/CCP17 FISH Probe (CytoTest). The sections of the slides were covered with coverslips and sealed with rubber glue. The probe and DNA in the sections were denatured in a ThermoBrite apparatus for 5 min at 75 °C, and then allowed to hybridize at 37 °C overnight. The cover slip was removed and the excess of non-hybridized and non-specifically bound probe was removed by washing the sections twice for 2 min each in post-hybridization washing buffer (2 × SSC/03% NP-40). After drying at room temperature, 10 μL of DAPI Solution was applied to the sections and the sections were covered again with a coverslip. The preparations were stored in a dark place until signal counting.

**Figure 1:**
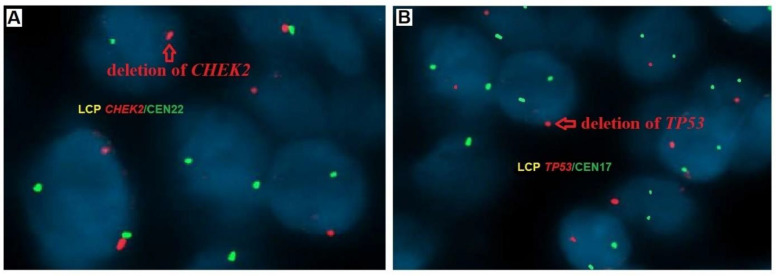
Gene status in PTC tumors samples examined by fluorescence in situ hybridization (FISH) using the probes LCP *CHEK2*/CEN22 and LCP TP53/CEN17; (**A**) detection of *CHEK2* (red signal indicates deletion of one copy of the *CHEK2* gene), and (**B**) detection of *TP53* (red signal indicates deletion of one copy of the *TP53* gene), magnification ×100.

*CHEK2*/CCP22 and TP53/CCP17 gene copy numbers were counted in 100 cell nuclei in non-overlapping fields of view containing cancer cells using a fluorescence microscope equipped with a double Orange/Green filter at 100× magnification with oil immersion. Hybridization with the *CHEK2*/CCP22 probe was confirmed by observing two red signals for the *CHEK2* and two green signals for the centromere of chromosome 22. Hybridization with the TP53/CCP17 probe was confirmed by observing two red signals for *TP53* and two green signals for the centromere of chromosome 17. The exact number of copies of each gene in each nucleus was recorded. When the copy number in more than 20% of the nuclei in each section exceeded the DNA ploidy number, this was considered gene amplification, whereas when the copy number in more than 30% of the nuclei was below the ploidy number, this was considered a gene deletion (Figure 1) [34].

### 2.5. Immunohistochemistry (IHC)

#### IHC Staining

Chk2(A-12) mouse monoclonal antibody from Santa Cruz Biotechnology (sc-5278) was used. Staining with this antibody was performed in an OMNIS staining device (Dako). Antigen unmasking was performed with EnV Flex TRS high pH reagent no. REF GV804 (Dako) at 97 °C for 20 min. The sections were incubated for 30 min with the primary antibody diluted 1:50 with EnV Flex/HRP polymer (Dako, REF GV800). 

For p53 mouse monoclonal antibody (DO7) (Cell Marque; REF 453M-95, catalog number CM-453M-95), staining was performed in an OMNIS staining device (Dako). Antigen unmasking was performed with EnV Flex TRS high pH reagent (Dako; no. REF GV804) at 97 °C for 20 min. The sections were incubated for 30 min with the primary antibody at a dilution of 1:500 with EnV Flex/HRP polymer (Dako, REF GV800).

### 2.6. Analysis of Immunohistochemical Results

**Figure 2:**
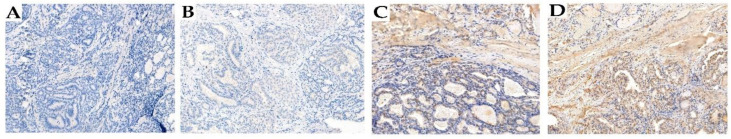
Detection of CHK2 expression by immunohistochemistry in PTC tumor samples. Representative staining intensities were scored as 0 (negative) (**A**), 1+ (weak) (**B**), 2+ (moderate) (**C**), and 3+ (strong) (**D**). Magnification ×200.

Immunohistochemical evaluation of CHK2 and p53 expression was performed by two pathologists specialized in PTC. The age of the tissue block had no effect on immunohistochemical staining. *CHEK2* staining was scored to evaluate the intensity of nuclear staining and determine the percentage of positive cells. Criteria for assessing the number of tumor cells were as follows: 0, no staining; 1, weak staining (≤10% of cells); 2, moderate staining (10–50% of cells); and 3, strong staining (>50% cancer cells). For statistical analysis, scores of 0 and 1 were classified as low CHK2 staining and scores of 2 and 3 as high staining. The following scoring system was also used for p53. Cases scored as positive showed strong nuclear staining in >30% of the cells, whereas normal thyroid follicular cells showed little staining. The CHK2 expression patterns revealed by IHC staining are shown in Figure 2.

### 2.7. Statistical Analyses

Continuous data are presented as the mean (standard deviation) and median (lower/upper quartiles and ranges (minimum and maximum)). Categorical data are presented as numbers and percentages. Odds ratio (OR) and 95% confidence intervals (95% CI) were calculated using logistic regression models. A two-tailed *p*-value < 0.05 was considered statistically significant. Two binomial proportions (e.g., CHK2 frequency in patients with and without vascular invasion) were compared using a Bayesian A/B test. The predictive performances of three competing hypotheses were examined. The first, the null hypothesis (H0), which was assigned a prior probability model (P(M)) of 0.50, predicted the equality of the tested binomial proportions and had a log OR = 0. The second, one-sided alternative hypothesis with an assigned P(M) of 0.25 predicted a higher percentage of results defined as “categories of interest” in the group of patients with the tested feature present (e.g., a higher percentage of patients without vascular invasion in the group of patients with the CHK2 present). The hypothesis with a log OR > 0 predicted a clinically beneficial effect of the tested feature. The third, complementary alternative hypothesis (log OR < 0 and P(M) = 0.25) predicted fewer successes in the selected group of patients, i.e., a clinically unfavorable impact of the tested feature. The results of testing the hypothesis are presented in the form of the Bayes factor (BF). The BF defines the ratio of two competing statistical models represented by their marginal probability. The BF was used to quantify the support for one model over another. The subscript used in BF notation indicated which hypothesis was supported by the data. BF_10_ indicated a BF that was more in favor of the alternative hypothesis than the H0 hypothesis. BF assumed that any positive value was qualitatively interpreted in accordance with the classification proposed by Jeffreys, modified by Lee et al. [35]. The prior and posterior densities of the log OR for the quantity of interest were plotted. A standard normal prior distribution (µ = 0 and σ = 1) on the log OR scale was adopted. The results were expressed as medians with 95% credible intervals (95% CI). For simplicity, descriptions of the results in the text were expressed as medians of the OR (anti-logarithm of log OR). A sequential analysis was performed to consider the results as a function of the total number of observations. The reproducibility of the estimates was determined by setting an initial random seed value (set seed = 123). All statistical analyses were performed using JASP (Version 16.0.0).

## 3. Results

### 3.1. Baseline Characteristics

Clinicopathological features of patients with PTC, molecular status of *CHEK2* mutation, primary treatment response categories, and disease outcomes for all 156 cases are presented in Table 1. In the study group of 156 Caucasian patients (median age was 49.5, range 39–59), the majority were women 138 (88.5%) and 98 patients were under 55 years of age (62.8%). The mean and standard deviation of tumor diameter for all patients was 13.1 ± 13.0 (range 1.0–84.0 mm). The stage of the primary lesion was pT1a in 94 (60.3%) patients, the dominant classic subtype was found in 107 (68.6%), gross extrathyroidal extension in two (1.3%), vascular invasion in 11 (7.1%), R1 surgical margin in 19 (12.2%), histologically verified lymph node metastases in 35 (22.4%), distant metastases in one (0.6%), and multifocality in 50 (32.1%) patients. Mutations in the *CHEK2* gene were found in 104 (66.7%) patients, including the I157T missense mutation in 60 (38.5%) and a truncating mutation (1100delC, IVS2 + 1G > A, del5395) in 38 (24.4%). No mutations in the *CHEK2* gene were found in 52 (33.3%) patients. Thirty-four patients (21.8%) were classified as intermediate risk and 25 patients (16.0%) as high risk according to ATA guidelines [27]. I-131 radiotherapy was administered to 111 (71.2%) patients, and 1100–3700 MBq of I-131 was used depending on cancer stage according to the TNM classification. Eighteen (11.5%) patients received I-131 radiotherapy more than once. The remaining 45 patients (28.9%) with a single PTC focus (≤1 cm in diameter) limited to the thyroid gland, without nodal and distant metastases (pT1aN0-xM0), did not receive I-131 treatment. In the entire study group, a very good response to primary treatment was observed in 127 (81.4%) patients. The median follow-up of the study group was 7 years (range 1–23). There were no cases of death due to cancer. However, deaths from other causes were recorded in five (3.2%) patients. At the end of follow-up, seven (4.5%) patients had an indeterminate response, one (0.6%) had biochemically persistent disease, and three (1.9%) had structurally persistent disease. Recurrence after a disease-free period (NED) occurred in five patients (3.2%).

#### 3.1.1. CHK2 and p53 Expression: Impact on Clinicopathological Features, Treatment Response and Outcomes in PTC Patients with and without CHEK2 Mutations

The relationship between CHK2 and p53 expression levels and clinicopathological features, response to treatment, and disease outcomes in 156 patients with PTC is presented in Table 2. There were no significant associations between CHK2 and p53 expression levels and sex, age at diagnosis, tumor size, histopathological subtypes, multifocality, lymph node (LN) metastases, distant metastases, extrathyroidal extension, vascular invasion, margin status, higher clinical stage of cancer, and intermediate and high recurrence or I-131 treatment. Patients with high CHK2 expression had a 70% and 80% lower chance of excellent and NED responses, respectively, than patients with no or lower CHK2 expression (OR, 0.28; 95% CI, 0.11–0.72; *p* = 0.009 and OR, 0.17; 95% CI, 0.05–0.62; *p* = 0.007). No such relationship was observed in patients who were positive for p53 expression.

#### 3.1.2. Impact of CHEK2 and TP53 Gene Status, and p53 Expression on Clinicopathological Features, Treatment Response and Outcomes in PTC Patients with and without CHEK2 Mutations

The relationships between clinicopathological features, response to treatment, and disease outcome in 151 patients with PTC (five patients were not included in the analysis due to degraded histopathological material) and *CHEK2* gene copy number, *TP53* gene copy number, and p53 expression together with *TP53* gene copy status are presented in Table 2. Positive p53 together with a *TP53* gene copy deletion was significantly associated with older patient age (≥55 years) at diagnosis (*p* = 0.047). A significant relationship was found between a *TP53* gene copy deletion and angioinvasion (*p* = 0.008). However, there was no significant relationship between *CHEK2* gene copy number, *TP53* gene copy number, and positive p53 together with a *TP53* gene copy deletion and clinicopathological features, response to treatment, and disease outcome.

#### 3.1.3. Prognostically Favorable and Predictive Results Defined as Categories of Interest in Groups of PTC Patients with and without CHEK2 Mutations

In the analyses described above, the associations between clinicopathological features (tumor diameter, papillary cancer histologic variant, vascular invasion, and age), response to therapy, and final follow-up and CHK2 expression, *TP53* gene copy number, p53 expression, and p53 expression together with *TP53* gene copy number were either statistically significant or borderline significant. These associations were further explored using Bayesian A/B testing (Table 3). Excellent response, NED in final follow-up, tumor size ≤10 mm (vs. >10 mm and ≤20 mm), and classic cancer subtype (vs. other aggressive subtypes) were ~1.4-fold, ~1.2-fold, ~1.4-fold, and ~1.1-fold higher, respectively, in patients with low CHK2 expression than in those with high CHK2 expression. In patients with no loss of a *TP53* gene copy and negative p53 together with no loss of a *TP53* gene copy, the absence of vascular invasion was ~1.4-fold and ~1.2-fold higher, respectively, than in patients with a *TP53* gene copy deletion and positive p53 together with a *TP53* gene copy deletion. In patients under 55 years of age, no loss of a *TP53* gene copy and negative p53 together with no loss of a *TP53* gene copy were observed ~2.0 and ~1.7 times more often, respectively.

#### 3.1.4. Predictive Value of Clinicopathological Features, Treatment Response, and Outcome Correlations with CHK2, TP53 Status, and p53 Expression in PTC Patients with and without CHEK2 Mutations

For all tested relationships (Table S1), H0 and the hypothesis predicting an increased frequency of categories defined as “categories of interest” in patients with high CHK2 expression, *TP53* gene copy deletion, and positive p53 together with a *TP53* gene copy deletion, the probability of the posterior model was reduced and these hypotheses were not consistent with the data. A third hypothesis predicting a reduced excellent response (compared to H0), NED in final follow-up, tumor size ≤10 mm (compared with >10 mm and ≤20 mm), classic cancer subtype (compared with other aggressive tumors), absence of vascular invasion, and age <55 years in patients with higher CHK2 expression, *TP53* gene copy deletion, and positive p53 together with a *TP53* gene copy deletion was qualitatively consistent with the data. The prior probability model of 0.25 was increased in all models from ~2-fold for positive p53 together with deletion of a *TP53* gene copy and age, and ~4-fold for higher CHK2 expression, treatment response, deletion of a *TP53* gene copy, and vascular invasion. In summary, the analyses provided strong or moderate evidence supporting the alternative hypothesis (compared with H0) predicting a lower prevalence of excellent response, NED in final follow-up, tumor size ≤10 mm (compared with >10 mm and ≤20 mm), classic cancer subtype (compared with other aggressive) in patients with higher CHK2 expression, lower prevalence of absence of vascular invasion, and age <55 years, in patients with deletion of the *TP53* gene copy and positive p53 together with deletion of the *TP53* gene copy. The accumulated evidence was 17,031, 20,554, 4766, and 4646 times more likely to support the hypothesis that excellent response, NED in final follow-up, tumor size ≤10 mm, and classic cancer subtype, respectively, occur less frequently in patients with higher CHK2 expression. The evidence was also 18,553 and 5713 times more likely to support the hypothesis that the absence of vascular invasion is less common in patients with a *TP53* gene copy deletion and positive p53 together with a *TP53* gene copy deletion, respectively. In patients with a *TP53* gene copy deletion and positive p53 together with a *TP53* gene copy deletion, the probability of being <55 years old was 4280 and 4962 times less likely than for patients without a *TP53* gene copy deletion and negative p53 together with no loss of *TP53* gene copy (Table S1).

#### 3.1.5. Posterior Distribution of Odds Log Ratio for Clinicopathological Correlations with CHK2, TP53 Status, and p53 Expression in PTC Patients with and without CHEK2 Mutations

Based on the median posterior distribution of the log OR, we found that in patients with high CHK2 expression compared with patients with low CHK2 expression, the probabilities of an excellent response, NED in final follow-up, tumor size ≤10 mm, and classic cancer subtype were 0.34 to 1, 0.27 to 1, 0.43 to 1, and 0.34 to 1, respectively. In patients with a *TP53* gene copy deletion and positive p53 together with a *TP53* gene copy deletion, the probabilities of not having vascular invasion were 0.23 to 1 and 0.35 to 1, respectively, compared with patients without these features. The probabilities of being <55 years of age in patients with a *TP53* gene copy deletion and positive p53 together with a *TP53* gene copy deletion were 0.39 to 1 and 0.42 to 1, respectively, compared with patients without this feature (Figure S1).

#### 3.1.6. Sequential Analysis of Clinicopathological Correlations with CHK2, TP53 Status, and p53 Expression in PTC Tumors with and without CHEK2 Mutations

All panel figures (Figure S2) indicated an increase in the likelihood of the alternative hypothesis predicting a reduced excellent response, NED in final follow-up, tumor size ≤10 mm, and classic cancer subtype in patients with higher CHK2 expression, lower absence of vascular invasion, and age <55 years in patients with *TP53* gene deletion and positive p53 together with *TP53* gene deletion.

#### 3.1.7. Correlation of Germline CHEK2 Mutation with p53 and CHK2 Expression, and CHEK2 and TP53 Gene Statuses in PTC Patients with and without CHEK2 Mutations

Table 4 shows the association between the germline *CHEK2* mutation and p53 expression, CHK2 expression, *CHEK2* and *TP53* gene statuses, and p53 expression together with *TP53* gene status. The association between germline heterozygous truncating *CHEK2* mutation variants (1100delC, IVS2 + 1G > A, del5395) with a *CHEK2* gene copy deletion and positive p53 together with deletion of a *TP53* gene copy was statistically significant. The chance of finding the *CHEK2* gene copy deletion in patients with germline heterozygous truncating *CHEK2* mutation variants (1100delC, IVS2 + 1G > A, del5395) was 62- and 57-fold higher than in patients with the WT *CHEK2* or WT *CHEK2* and patients with the missense I157T mutation, respectively. The chance of finding positive p53 together with a *TP53* gene copy deletion in patients with germline heterozygous truncating *CHEK2* mutation variants (1100delC, IVS2 + 1G > A, del5395) was more than 3-fold higher than in patients with WT *CHEK2* and the missense I157T mutation combined. Other associations between germline *CHEK2* mutations and p53 expression, CHK2 expression, *CHEK2* gene copy status, p53 expression together with *TP53* gene copy status, and *TP53* gene copy status alone were not statistically significant. 

#### 3.1.8. The Incidence of Unfavorable Prognostic Factors Linked to CHEK2 and TP53 Gene Alterations, CHK2 and p53 Expression in PTC Patients with and without CHEK2 Mutations

The analyses described above examined the associations between germline heterozygous truncating *CHEK2* mutation variants (1100delC, IVS2 + 1G > A, del5395) and *CHEK2* gene status, CHK2 expression, and p53 expression together with TP53 gene status. We decided to examine further the associations that were significant or marginally significant. We tested the equality of following two binomial proportions: *CHEK2* gene copy deletion/no deletion; higher/lower CHK2 expression, and positive/negative p53 together with *TP53* gene copy deletion/no deletion in patients with *CHEK2* truncating mutations (1100delC, IVS2 + 1G > A, del5395), and in patients with WT *CHEK2* or WT *CHEK2* and the missense I157T mutation using the Bayesian A/B test. In the compared groups, categories of interest were defined as *CHEK2* gene copy deletion, higher CHK2 expression, and positive p53 together with a TP53 gene copy deletion. The results are presented in Table 5. Among patients with germline heterozygous truncating *CHEK2* mutation variants (1100delC, IVS2 + 1G > A, del5395) the percentages of cases with a *CHEK2* gene copy deletion, higher CHK2 expression, and positive p53 together a *TP53* gene copy deletion were ~10-, ~3-, and ≈3-fold higher than in those with WT *CHEK2*, respectively. Among patients with germline heterozygous truncating *CHEK2* mutation variants (1100delC, IVS2 + 1G > A, del5395) and the missense I157T mutation, the percentages of cases with a *CHEK2* gene copy deletion, higher CHK2 expression, and positive p53 together with a *TP53* gene copy deletion were ~10-, ~2-, and ~3-fold higher, respectively, than in those with WT *CHEK2* or the missense I157T mutation (Table 5). 

#### 3.1.9. Predictive Value of CHEK2 and TP53 Gene Status, CHK2 and p53 Expression in PTC Patients with WT and/or I157T CHEK2 Mutations

Table S2 shows the predictive value of the three competing hypotheses. For H0 and the hypothesis predicting no *CHEK2* gene copy deletion, low CHK2 expression, and negative p53 together with no loss of TP53 gene copy in patients with germline heterozygous *CHEK2* truncating mutations (1100delC, IVS2 + 1G > A, del5395) compared with those with WT *CHEK2* or WT *CHEK2* and those with the missense I157T mutation, posterior model probabilities were reduced (compared to prior model probabilities) and these hypotheses were not consistent with the data. The third hypothesis predicted higher probabilities of a *CHEK2* gene copy deletion, high CHK2 expression, and positive p53 together with deletion of the *TP53* gene copy in patients with heterozygous *CHEK2* truncating germline mutations (1100delC, IVS2 + 1G > A, del5395) than in those with WT *CHEK2* or WT *CHEK2* and those with the missense I157T mutation. The predictions were qualitatively consistent with the data; the a priori model probability of 0.25 was increased by more than 4-fold in both tested models for the *CHEK2* gene copy deletion, 2-fold in both models for high CHK2 expression, and 2- and 3-fold in models for positive p53 together with deletion of the *TP53* gene copy. To sum up, the analyses provided high or moderate evidence supporting the alternative hypothesis (compared with H0) predicting higher probabilities of a *CHEK2* gene copy deletion, high CHK2 expression, and positive p53 together with a *TP53* gene copy deletion in patients with the heterozygous *CHEK2* truncating germline mutation (1100delC, IVS2 + 1G > A, del5395) than in those with WT *CHEK2* or WT *CHEK2* and those with the I157T missense mutation, with the accumulated evidence showing that the alternative hypothesis (log OR > 0) would predict that these features are 1781 × 10^10^, 4215, and 3194 times more likely, respectively, in patients with *CHEK2* truncating germline mutations (1100delC, IVS2 + 1G > A, del5395) than in those with WT *CHEK2*. The evidence also showed that it was 1054 + 15, 3058, and 9999 times more likely that the alternative hypothesis (log OR > 0) would predict that the *CHEK2* gene copy deletion, high CHK2 expression, and positive p53 with a *TP53* gene copy deletion, respectively, occurs more often in patients with heterozygous *CHEK2* truncating germline mutations (1100delC, IVS2 + 1G > A, del5395) than in those with WT *CHEK2* and the missense I157T mutation.

#### 3.1.10. Posterior Odds Log Ratio Distribution for CHEK2 and TP53 Gene Status, CHK2 and p53 Expression in PTC Patients with and without CHEK2 Mutations

Based on the median posterior distribution for the log OR, we found that the probability of a *CHEK2* gene copy deletion in patients with germline heterozygous *CHEK2* truncating mutations (1100delC, IVS2 + 1G > A, del5395) was ~22 to 1 and ~26 to 1, respectively, compared with patients with WT *CHEK2* and WT *CHEK2* with the missense I157T mutation. The probabilities of higher CHK2 and positive p53 in the presence of *TP53* gene copy deletion in patients with a *CHEK2* truncating germline mutation (1100delC, IVS2 + 1G > A, del5395) were ~2.5 to 1 and ~2 to 1, respectively, compared with patients with WT *CHEK2*, whereas the probabilities were ~2 to 1 and ~3 to 1 compared with patients with WT *CHEK2* and WT *CHEK2* with the missense I157T mutation, respectively (Figure S3).

#### 3.1.11. Sequential Analysis of Associations Involving CHEK2 and TP53 Gene Status, CHK2 and p53 Expression in PTC Patients with and without CHEK2 Mutations

Based on the sequential analysis, the obtained results were considered as a function of the total number of observations. The panels in Figure S4 depict two circles visualizing the prior and posterior probabilities of the tested hypotheses based on all available data. All the panels in the figures indicate an increase in the probability of the alternative hypothesis that *CHEK2* gene copy deletion, higher CHK2 expression, or positive p53 together with the *TP53* gene copy deletion occur more frequently in patients with a germline heterozygous *CHEK2* truncating mutation (1100delC, IVS2 + 1G > A, del5395) (Figure S4). 

## 4. Discussion

Cell cycle checkpoints induced by DNA mutations play a key role in maintaining genome stability. Tumor suppressors associated with DNA damage responses and the regulation of cell cycle checkpoints include checkpoint kinase 2 (*CHEK2* gene, CHK2) and p53. Due to defects in the function of suppressor genes involved in the DNA repair pathway, genome instability and a predisposition to cancer development may occur [6,36]. Depending on the nature of the damage, the type and function of the damaged cell, and other factors, CHK2 can phosphorylate over 20 different proteins involved in the regulation of cellular responses, including p53 and BRCA1 [37]. The association between germline mutations in the *CHEK2* gene and the development of various types of cancer, including PTC, has been reported in numerous case-control studies [20,38,39,40,41,42,43,44,45,46]. To date, the mechanism responsible for the increased CHK2 levels in PTC patients remains poorly understood. The data in this study identified CHK2 expression as an independent prognostic parameter of PTC. Patients expressing CHK2 had a 70% and 80% lower chance of an excellent response and NED, respectively, than patients not expressing or expressing lower amounts of CHK2. Tumor size, histological subtype of the cancer (features that were of borderline statistical significance), response to treatment, and final disease status (features that were statistically significant) were further explored using the Bayesian A/B test. 

Excellent response to primary treatment, final follow-up NED, tumor size ≤ 10 mm (compared with >10 mm and ≤20 mm), and classic cancer type (compared with other aggressive subtypes) were from 10% to 40% higher in patients without CHK2 expression than in those with CHK2 expression. In a study by Zhao W et al. 2018 [10], it was found that CHK2 expression levels and phosphorylated CHK2 (p-CHK2) were significantly higher in most PTCs than in tumor-adjacent thyroid tissues. They also reported that high p-CHK2 expression, male sex, classical variant, stage N, and LN metastasis were associated with cancer aggressiveness. In the present study, we did not evaluate p-CHK2 expression. However, CHK2 expression in the study by Zhao W et al. 2018 correlated significantly and negatively with multifocality [10], but we were unable to evaluate this in the present study. Zhao W et al. 2018 also found that CHK2 and p-CHK2 expression were significantly downregulated in LN metastasis compared with matched primary PTC tumors and suggested that CHK2 levels may be negatively associated with circulating tumor cell survival and metastatic behavior [10]. In the current study, we also analyzed CHK2 expression in all 35 patients with LN metastasis, but found that CHK2 expression in LN metastasis tumors was not higher than that in matched primary tumors (Figure S5).

Similarly, increased CHK2 expression has been observed in other human malignancies, such as melanoma metastases, most cases of DLBCL [11], bladder cancer, and ovarian cancer [13]. However, other studies on the prognostic value of CHK2 expression revealed low CHK2 expression was associated with poor prognosis in 918 cases of gastric cancer. These differences in patient outcomes may be because CHK2 has multiple and diverse functions and interacts with a wide variety of key cancer genes, each of which can cause CHK2 overexpression under certain conditions [47,48].

The molecular database employed in the present study allowed us to examine the association between CHK2 expression and molecular features of interest. We selected patients with germline mutations in the *CHEK2* gene (truncating 1100delC, IVS2 + 1G > A, del5395, and missense I157T mutations) and patients without a mutation in the *CHEK2* gene (*CHEK2* WT). In our previous work, we showed that truncating mutations, but not missense mutations, in the *CHEK2* gene are significantly associated with angioinvasion and an intermediate or high initial risk of recurrent/persistent disease in PTC patients [21]; however, we did not evaluate CHK2 and p53 expression in tumor tissues. Therefore, in the current study, we performed IHC analysis of CHK2 and p53 expression in tumors to assess the value of the ATM/CHK2/p53 pathway as a prognostic marker. In addition, we used FISH to analyze for *CHEK2* and TP53 copy number aberrations and correlate these aberrations with the course of PTC disease. Positive p53 expression together with TP53 gene deletion was significantly associated with older (≥55 years) patient age at diagnosis. This can be attributed to the decrease in p53 pathway activity with age, which is associated with an increased mutation rate (due to impaired DNA repair) and mutation fixation (due to a decrease in p53-mediated apoptosis) in older people. The decline in p53 function with age may result in the accumulation of TP53 mutations, which is probably why older people have lower rates of cell division fidelity, higher DNA replication error rates, and a higher incidence of cancer [49]. In this study, we found a significant association between angioinvasion and TP53 gene copy deletion, but this association lost statistical significance when we examined the association between angioinvasion and positive p53 expression together with a TP53 gene copy deletion. These associations were further explored using a Bayesian A/B test, where patients with no loss of a TP53 gene copy together with negative p53 expression and patients with no loss of a TP53 gene copy alone had a 20% to 40% lower prevalence of vascular invasion than patients with no loss of a TP53 gene copy together with positive p53 expression and patients with a TP53 gene copy deletion alone. So far, angioinvasion is thought to be associated with an unfavorable clinical course and poor prognosis, which has important consequences for cancer treatment [50]. Moreover, in accordance with the ATA Management Guidelines criteria, vascular invasion is a key feature that is absent from low-risk PTC patients [27]. Mutations in the TP53 gene, in addition to poorly differentiated and anaplastic thyroid cancer, where such mutations occur most frequently, have also been found in some well-differentiated cancers, including PTC [51]. It is also likely that well-differentiated cancers with a TP53 mutation are more inclined to undergo tumor dedifferentiation and result in a more aggressive clinical course [52].

To the best of our knowledge, no previous studies have assessed CHK2 and p53 expression by IHC and *CHEK2* and TP53 gene statuses by FISH in a large group of PTC patients with germline mutations in the *CHEK2* gene. The only study to have evaluated patients with a germline *CHEK2* mutation in PTC patients was a paper by Zhao Y et al. [53], which reported a Chinese family with a germline *CHEK2* mutation c.417C > A. Molecular analysis showed that the 417C > A substitution created a premature stop codon (Y139X). Tumor tissue analysis showed that two patients with the loss-of-function Y139X variant exhibited lower mRNA expression of the mutant allele, lower CHK2 expression, and lower p53 and phosphorylated p53 expression than in patients with sporadic PTC (WT *CHEK2*). The molecular database employed in the present study comprised patients with truncating mutations (1100delC, IVS2 + 1G > A, and del5395) and a I157T missense mutation, but no patients with a nonsense mutation [53].

The association between CHK2 expression and mutation of the *CHEK2* gene was clear in patients with truncating mutations (1100delC, IVS2 + 1G > A, and del5395). In patients with *CHEK2* truncating mutations, one copy of the *CHEK2* gene remains intact (in the FISH method in this study, all detected deletions had only one damaged copy of the gene) while the other is truncated. The intact copy can synthesize a sufficient amount of CHK2, or in some cases produce larger amounts of CHK2 than cells with two normal copies of *CHEK2*. Complete loss of CHK2 function only occurs when the remaining intact copy is mutated or deleted, termed loss of heterozygosity, but we did not test for this in our study.

On the other hand, higher CHK2 expression may be due to impaired degradation of CHK2, the mechanism of which is not fully understood in cancer cells. It is possible that the ubiquitin-proteasome pathway was disturbed in the tumor cells. The ubiquitin-proteasome pathway is crucial for the expression and control of the activity of proteins involved in cell cycle regulation and the DNA damage response. It is known that interactions between proteins may change in response to DNA damage [54].

Increased expression of CHK2 may also be attributed to impaired ubiquitination caused by aberrations in p53. Bohgaki et al. demonstrated that, in mice, CHK2 phosphorylated at S460 (which corresponds to S456 in humans) undergoes ubiquitination that is facilitated by the p53-induced RING-H2 protein (PIRH2) and subsequent degradation by the proteasome [55].

Our study has several strengths. First, the study examined a large homogeneous group of Caucasian patients from one center in Poland. Second, to our knowledge, there have been no studies in the literature assessing CHK2 and p53 expression in a large number of PTC patients with a germline mutation in the *CHEK2* gene. Third, there have been no studies assessing the statuses of the *CHEK2* and TP53 genes by FISH in patients with PTC.

Our study has some limitations. First, the study mainly included low-risk tumors (62.2%), with a high number of microcarcinomas (≤1 cm, 60.3%), and this could have influenced the results. Second, the number of patients with advanced clinical disease was relatively small. Third, the follow-up period was short (7 years). Follow-up periods beyond 7 years could reveal more information about PTC-related recurrence or death and improve understanding of the roles of CHK2 and p53. Due to financial constraints, we were unable to examine the tumor tissues for phosphorylated CHK2 and p53 or sequence both genes (*CHEK2*, TP53) in the tumor tissues, which could have improved the risk stratification. It will be interesting to evaluate p-CHK2 and p53 expression and sequence both genes in future studies.

## 5. Conclusions

High CHK2 expression is associated with poor treatment response and outcomes in patients with PTC. Increased expression of CHK2 and positive p53 expression together with deletion of a TP53 gene copy in patients with a truncating mutation in the *CHEK2* gene could have potential as a prognostic marker for an unfavorable disease course in this group of patients. Therefore, further studies will be required using larger patient groups with germline mutations, in particular those with a truncating mutation in the *CHEK2* gene, to assess whether patients with increased expression of CHK2 and p53 in cancer cells, especially their phosphorylated forms, require different treatment regimens or oncological follow-up protocols from those of patients with tumors that do not express these genes.

## Figures and Tables

**Table 1:** Basic characteristics of 156 patients with papillary thyroid carcinoma.

Characteristic	Total *n* = 156 (100%)
Sex	
Female	138 (88.5%)
Male	18 (11.5%)
Age at diagnosis (years)	
<55	98 (62.8%)
≥55	58 (37.2%)
Mean (SD)	48.5 (13.6)
Median (Q1–Q3)	49.5 (39.0–59.0)
Range	18.0–73.0
Tumor diameter (mm)	
Mean (SD)	13.1 (13.0)
Median (Q1–Q3)	8.0 (5.0–16.5)
Range	1.0–84.0
Tumor diameter (mm)	
≤10	94 (60.3%)
>10–20	32 (20.5%)
>20–40	25 (16.0%)
>40	5 (3.2%)
Papillary cancer histologic variant	
Classic	107 (68.6%)
Follicular	35 (22.4%)
Oxyphilic	3 (1.9%)
Diffuse sclerosing	3 (1.9%)
Tall cell	1 (0.6%)
Other *	7 (4.5%)
Surgical treatment	
Total thyroidectomy	152 (97.4%)
Lobectomy	4 (2.6%)
Nodal dissection	
Central	94 (60.3%)
Lateral	17 (10.9%)
No dissection	45 (28.8%)
Multifocality	
No	106 (68.0%)
Yes	50 (32.1%)
Lymph node metastases **	
N0a	76 (48.7%)
N0b	45 (28.9%)
N1a	18 (11.5%)
N1b	17 (10.9%)
Distant metastases	
M0	155 (99.4%)
M1	1 (0.6%)
Extrathyroidal extension	
Negative	124 (79.5%)
Microscopic	30 (19.2%)
Gross	2 (1.3%)
Vascular invasion	
No	145 (93%)
Yes	11 (7.1%)
Margin status	
R0	137 (87.8%)
R1	19 (12.2%)
Tumor stage	
pT1a	94 (60.3%)
pT1b	32 (20.5%)
pT2	23 (14.7%)
pT3a	5 (3.2%)
pT3b	2 (1.3%)
*CHEK2* mutation status	
*CHEK2* WT	52 (33.3%)
*CHEK2* mutation any	104 (66.7%)
*CHEK2* I157T missense mutation (any)	60 (38.5%)
I157T heterozygous	53 (34.0%)
I157T homozygous	7 (4.5%)
*CHEK2* truncating heterozygous mutation (any)	38 (24.4%)
IVS2 + 1G > A	16 (10.3%)
Del5395	10 (6.4%)
1100delC	12 (7.7%)
Coexistence of two heterozygous *CHEK2* mutations	6 (3.8%)
I157T and IVS2 + 1G > A	3 (1.9%)
I157T and Del5395	1 (0.6%)
IVS2 + 1G > A and Del5395	2 (1.3%)
TNM (8th edition)	
I	144 (92.3%)
II	12 (7.7%)
ATA initial risk stratification system	
Low	97 (62.2%)
Intermediate	34 (21.8%)
High	25 (16.0%)
Radioactive iodine (I-131) therapy	
No	45 (28.9%)
Yes	111 (71.2%)
More than one course of radioactive iodine therapy (I-131)	
No	138 (88.5%)
Yes	18 (11.5%)
Response to therapy	
Excellent	127 (81.4%)
Indeterminate	24 (15.4%)
Biochemically incomplete	2 (1.3%)
Structurally incomplete	3 (1.9%)
Final follow-up (disease outcome)	
NED	145 (93.0%)
Indeterminate	7 (4.5%)
Biochemically persistent	1 (0.6%)
Structurally persistent	3 (1.9%)
Follow-up, recurrence	
No	151 (96.8%)
Yes	5 (3.2%)
Death	
No	151 (96.8%)
Yes	5 (3.2%)
Follow-up (years)	
Median (range)	7.0 (1.0–23.0)

* Warthin-like (*n* = 2); mixed variant (classic and follicular) (*n* = 5). ** N0a, one or more cytologically or histologically confirmed benign lymph nodes; N0b, no radiologic or clinical evidence of locoregional lymph node metastasis; N1a–N1b, metastasis to regional lymph nodes; R1, microscopically positive margin (resection); ATA, American Thyroid Association, determined according to the 8th edition of the American Joint Committee on Cancer/Union for International Cancer Control (tumor-node-metastasis) TNM staging system; *CHEK2* WT (wild type), cases without the following mutations: I157T, 1100delC, IVS2 + 1G > A, del5395; SD, standard deviation; NED, no evidence of disease.

**Table 2:** Relationship between clinicopathological characteristics and p53 expression, CHK2 expression, *CHEK2* gene copy deletion, *TP53* deletion, and p53 expression together with TP53 deletion in the tumor tissues of patients with and without germline *CHEK2* mutations.

Variable	p53, *n* = 156 (100%)		CHK2, *n* = 156 (100%)		*CHEK2* (FISH), *n* = 151 (100%)		TP53 (FISH), *n* = 151 (100%)		Positive p53 with TP53 Deletion (FISH)	
	Positive *n* = 8 (5.1%)		High Expression *n* = 23 (14.7%)		Deletion *n* = 44 (29.1%)		Deletion *n* = 7 (4.6%)		*n* = 15 out of 151 (9.9%)	
	OR (95% CI)	*p*	OR (95% CI)	*p*	OR (95% CI)	*p*	OR (95% CI)	*p*	OR (95% CI)	*p*
Sex										
Female vs. male	NA (0 in cell)		1.44 (0.31–6.71)	0.645	1.08 (0.36–3.23)	0.892	1.09 (0.13–9.24)	0.938	NA (0 in cell)	
Age group										
≥55 years vs. <55 years	2.99 (0.69–13.00)	0.145	0.55 (0.20–1.49)	0.238	0.75 (0.36–1.58)	0.451	3.92 (0.94–16.35)	0.061	3.03 (1.02–9.05)	0.047
Tumor stage										
pT1a vs. pT1b, pT2, pT3a, pT3b	NA (0 in cell)		0.96 (0.11–8.38)	0.972	0.97 (0.18–5.21)	0.973	0.81 (0.21–3.16)	0.766	0.99 (0.33–2.93)	0.982
TNM (8th edition)										
TNM II vs. TNM I	NA (0 in cell)		1.17 (0.24–5.73)	0.845	0.52 (0.11–2.50)	0.414	1.65 (0.19–14.54)	0.652	0.90 (0.11–7.56)	0.923
Multifocality										
Yes vs. no	0.69 (0.14–3.57)	0.662	0.92 (0.35–2.39)	0.857	1.00 (0.47–2.13)	0.996	0.62 (0.12–3.08)	0.555	0.52 (0.14–1.95)	0.334
Lymph node metastasis *										
N1a, N1b vs. N0b, N0a	NA (0 in cell)		0.95 (0.33–2.78)	0.931	1.02 (0.44–2.35)	0.968	0.40 (0.05–3.29)	0.392	0.21 (0.03–1.69)	0.144
Distant metastasis										
M1 vs. M0	NA (0 in cell)		NA (0 in cell)		NA (0 in cell)		NA (0 in cell)	0.993	NA (0 in cell)	
Tumor diameter										
>10 and ≤20 mm vs. ≤10 mm	1.84 (0.41–8.18)	0.422	2.80 (1.00–7.88)	0.051	0.78 (0.31–1.95)	0.592	1.84 (0.41–8.21)	0.422	1.75 (0.54–5.70)	0.351
>20 and ≤40 mm vs. ≤10 mm	NA (0 in cell)		2.10 (0.65–6.83)	0.217	1.31 (0.52–3.34)	0.568	0.72 (0.08–6.43)	0.766	0.38 (0.05–3.15)	0.370
>40 mm vs. ≤10 mm	NA (0 in cell)		NA (0 in cell)		NA (0 in cell)		NA (0 in cell)		NA (0 in cell)	
Papillary cancer histologic variant										
Follicular vs. Classic	1.24 (0.23–6.68)	0.805	1.02 (0.34–3.05)	0.969	1.24 (0.54–2.86)	0.618	0.44 (0.05–3.69)	0.447	0.95 (0.25–3.68)	0.941
Other ** aggressive vs. Classic	NA (0 in cell)		6.13 (0.80–46.91)	0.081	0.86 (0.09–8.63)	0.900	4.67 (0.43–50.91)	0.206	3.17 (0.30–33.37)	0.337
Other *** non-aggressive vs. Classic	2.27 (0.24–21.56)	0.476	0.68 (0.08–5.77)	0.725	1.29 (0.30–5.52)	0.728	NA (0 in cell)		1.19 (0.13–10.49)	0.877
Extrathyroidal extension										
Micro and gross vs. negative	1.31 (0.25–6.83)	0.748	1.09 (0.37–3.20)	0.875	1.45 (0.63–3.35)	0.385	1.11 (0.22–5.65)	0.897	0.68 (0.20–2.31)	0.537
Vascular invasion										
Yes vs. no	NA (0 in cell)		1.31 (0.27–6.50)	0.739	2.62 (0.72–9.53)	0.145	8.38 (1.76–39.80)	0.008	4.00 (0.94–17.10)	0.061
Margin status										
R1 vs. R0	NA (0 in cell)		0.65 (0.14–3.02)	0.583	0.85 (0.29–2.53)	0.772	0.92 (0.11–7.81)	0.938	0.50 (0.06–4.05)	0.516
ATA										
Low vs intermediate, high	1.01 (0.23–4.41)	0.985	0.62 (0.25–1.50)	0.287	1.05 (0.50–2.17)	0.906	0.72 (0.19–2.81)	0.639	1.55 (0.53–4.54)	0.421
Response to therapy										
Excellent vs. others	NA (0 in cell)		0.28 (0.11–0.72)	0.009	0.85 (0.34–2.14)	0.731	1.63 (0.19–13.61)	0.653	0.33 (0.04–2.66)	0.300
Radioactive iodine (I-131) therapy										
Yes vs. no	0.66 (0.15–2.89)	0.581	2.12 (0.68–6.61)	0.197	0.72 (0.34–1.53)	0.391	3.48 (0.42–28.64)	0.247	1.15 (0.34–3.81)	0.824
Number of I-131 courses										
2–9 courses vs. 0–1 course	2.75 (0.51–14.79)	0.239	0.70 (0.15–3.25)	0.646	2.16 (0.79–5.89)	0.134	0.92 (0.11–7.81)	0.938	2.02 (0.51–7.97)	0.317
Final follow-up										
NED vs. others	NA (0 in cell)		0.17 (0.05–0.62)	0.007	0.96 (0.24–3.88)	0.951	0.54 (0.06–4.82)	0.582	0.99 (0.12–8.42)	0.994
Follow-up recurrence										
Yes vs. no	NA (0 in cell)		1.47 (0.16–13.74)	0.738	NA (0 in cell)		NA (0 in cell)		NA (0 in cell)	
Death										
Yes vs. no	NA (0 in cell)		NA (0 in cell)		0.60 (0.07–5.51)	0.651	4.31 (0.43–43.20)	0.214	2.36 (0.25–22.58)	0.457

* N0a, one or more cytologically or histologically confirmed benign lymph nodes; N0b, no radiologic or clinical evidence of locoregional lymph node metastasis; N1a–N1b, metastasis to regional lymph nodes; **diffuse sclerosing (n = 3); tall cell variant (n = 1); *** oxyphilic, Warthin-like (n = 2); mixed variant (classic and follicular) (n = 5); R1-microscopically positive margin (resection); ATA, American Thyroid Association, determined according to the 8th edition of the American Joint Committee on Cancer/Union for International Cancer Control (tumor-node-metastasis) TNM staging system; NED, no evidence of disease.

**Table 3:** The prevalence of prognostically favorable and predictive results, defined as category of interest in groups of PTC patients with and without *CHEK2* germline mutations.

Group	Category of Interest	Counts	Total	Proportion
Response to therapy	Excellent			
Low CHK2		113	133	0.850
High CHK2		14	23	0.609
Final follow-up	NED			
Low CHK2		127	133	0.955
High CHK2		18	23	0.783
Tumor diameter	≤10 mm			
Low CHK2		84	108	0.788
High CHK2		10	18	0.556
Papillary cancer histologic variant	Classic			
Low CHK2		92	94	0.979
High CHK2		15	17	0.882
Age	<55 years			
No loss of TP53		94	142	0.662
Deletion of TP53		3	9	0.333
Vascular invasion	No			
No loss of TP53		134	142	0.944
Deletion of TP53		6	9	0.667
Age	<55 years			
Negative p53 + no loss of TP53		91	136	0.669
Positive p53 + Deletion of TP53		6	15	0.400
Vascular invasion	No			
Negative p53 + no loss of TP53		128	136	0.941
Positive p53 + Deletion of TP53		12	15	0.800

CHK2 loss/low/high, CHK2 loss/low/high expression as determined by IHC on tumor tissue; IHC, immunohistochemistry; no loss of TP53/deletion of TP53, no loss/deletion of gene copy in tumor tissue as determined by FISH; FISH, fluorescence in situ hybridization; NED, no evidence of disease.

**Table 4:** Relationships between the germline *CHEK2* mutation and p53 expression, CHK2 expression, *CHEK2* and *TP53* gene statuses, and p53 expression together with *TP53* gene status (FISH) in tumor tissues from PTC patients with and without *CHEK2* germline mutations.

Variable	*CHEK2* Truncating vs. WT		*CHEK2* Missense vs. WT		*CHEK2* Truncating vs. WT + Missense I157T	
	OR (95% CI)	*p*	OR (95% CI)	*p*	OR (95% CI)	*p*
p53 expression						
Positive vs. negative	0.42 (0.04–4.19)	0.459	1.17 (0.25–5.47)	0.845	0.35 (0.04–2.92)	0.331
CHK2 expression						
High vs. low	3.48 (0.99–12.30)	0.052	2.12 (0.61–7.33)	0.236	2.24 (0.90–5.58)	0.083
*CHEK2* gene status						
Deletion vs. no loss of gene copy	62.67 (16.37–239.93)	<0.001	1.31 (0.35–4.91)	0.693	57.23 (19.36–169.24)	<0.001
*TP53* gene status						
Deletion vs. no loss of gene copy	NA (0 in cell)		NA (0 in cell)		NA (0 in cell)	
p53 expression and *TP53* gene status						
Positive vs. negative and deletion vs. no loss of gene copy	3.47 (0.84–14.37)	0.087	1.17 (0.25–5.47)	0.845	3.33 (1.13–9.84)	0.029

Abbreviations: *CHEK2* truncating, germline heterozygous truncating *CHEK2* mutation variants (1100delC, IVS2 + 1G > A, del5395); WT, wild type.

**Table 5:** The incidence of unfavorable prognostic factors, such as deletion of the *CHEK2* gene copy, higher CHK2 expression, and p53 expression together with deletion of the *TP53* gene copy by FISH, in tumor tissues from PTC patients with and without the *CHEK2* germline mutations.

Variable	*CHEK2* Truncating and WT				*CHEK2* Truncating and WT + Missense I157T		
	Group	Counts	Total	Proportion	Counts	Total	Proportion
*CHEK2* deletion	No mutation	4	51	0.078	10	111	0.090
	Mutation	32	38	0.842	34	40	0.850
High expression of CHK2	No mutation	4	52	0.077	13	112	0.116
	Mutation	9	40	0.225	10	44	0.227
Positive p53 and *TP53* deletion	No mutation	3	51	0.059	7	111	0.063
	Mutation	7	38	0.184	8	40	0.200

Abbreviations: *TP53* deletion, deletion of a TP53 gene copy in tumor tissue as determined by FISH; FISH, fluorescence in situ hybridization; positive p53, p53 expression as determined by IHC; IHC, immunohistochemistry; *CHEK2* truncating, germline heterozygous truncating *CHEK2* mutation variants (1100delC, IVS2 + 1G > A, del5395); WT, wild type.

## Data Availability

Data are available on reasonable request.

## Supplementary Materials

The following supporting information can be downloaded at: https://www.mdpi.com/article/10.3390/cancers16040815/s1, Figure S1: Posterior distribution of the odds log ratio for the relationships between clinicopathological features and CHK2 expression, TP53 gene status, and p53 expression together with TP53 gene status in tumor tissues from PTC patients with and without CHEK2 germline mutations; Figure S2: Sequential analysis for the relationships between clinicopathological features and CHK2 expression, TP53 gene status, and p53 expression together with TP53 gene status in the tumor tissues of PTC patients with and without CHEK2 germline mutations; Figure S3: Posterior distribution of the odds log ratio for the association between CHEK2 gene status, CHK2 expression, and the p53 expression together with TP53 gene status in tumor tissues from PTC patients with and without the CHEK2 germline mutations; Figure S4: Sequential analysis of the associations between CHEK2 gene status, CHK2 expression as determined by IHC, and p53 expression together with TP53 gene status as determined by FISH in tumor tissues from PTC patients with and without the CHEK2 germline mutations; Figure S5: CHK2 expression levels in primary papillary thyroid cancer tissue and lymph node metastases; Table S1: Predictive values of the tested hypotheses for the relationships between clinicopathological features, treatment response, and disease outcome and level of CHK2 expression and TP53 gene status, and p53 expression together with TP53 status in tumor tissues from PTC patients with and without CHEK2 germline mutations; Table S2: Predictive value of tested hypotheses for the association between CHEK2 gene status, CHK2 expression, and p53 expression together with TP53 gene status in PTC patients with and without the CHEK2 germline mutations.
