# Supplementary material for: Relationship between the Expression of CHK2 and p53 in Tumor Tissue and the Course of Papillary Thyroid Cancer in Patients with CHEK2 Germline Mutations
Source: Cancers (Basel). 2024 Feb 17;16(4):815. doi: 10.3390/cancers16040815 (PMC10886656; doi:10.3390/cancers16040815)
Supplement: Supplementary file 1 [file cancers-16-00815-s001.zip › cancers-2823324-supplementary.pdf]

Figure S1: Posterior distribution of the odds log ratio for the relationships between clinicopathological features and CHK2 expression, *TP53* gene status, and p53 expression together with *TP53* gene status in tumor tissues from PTC patients with and without *CHEK2* germline mutations

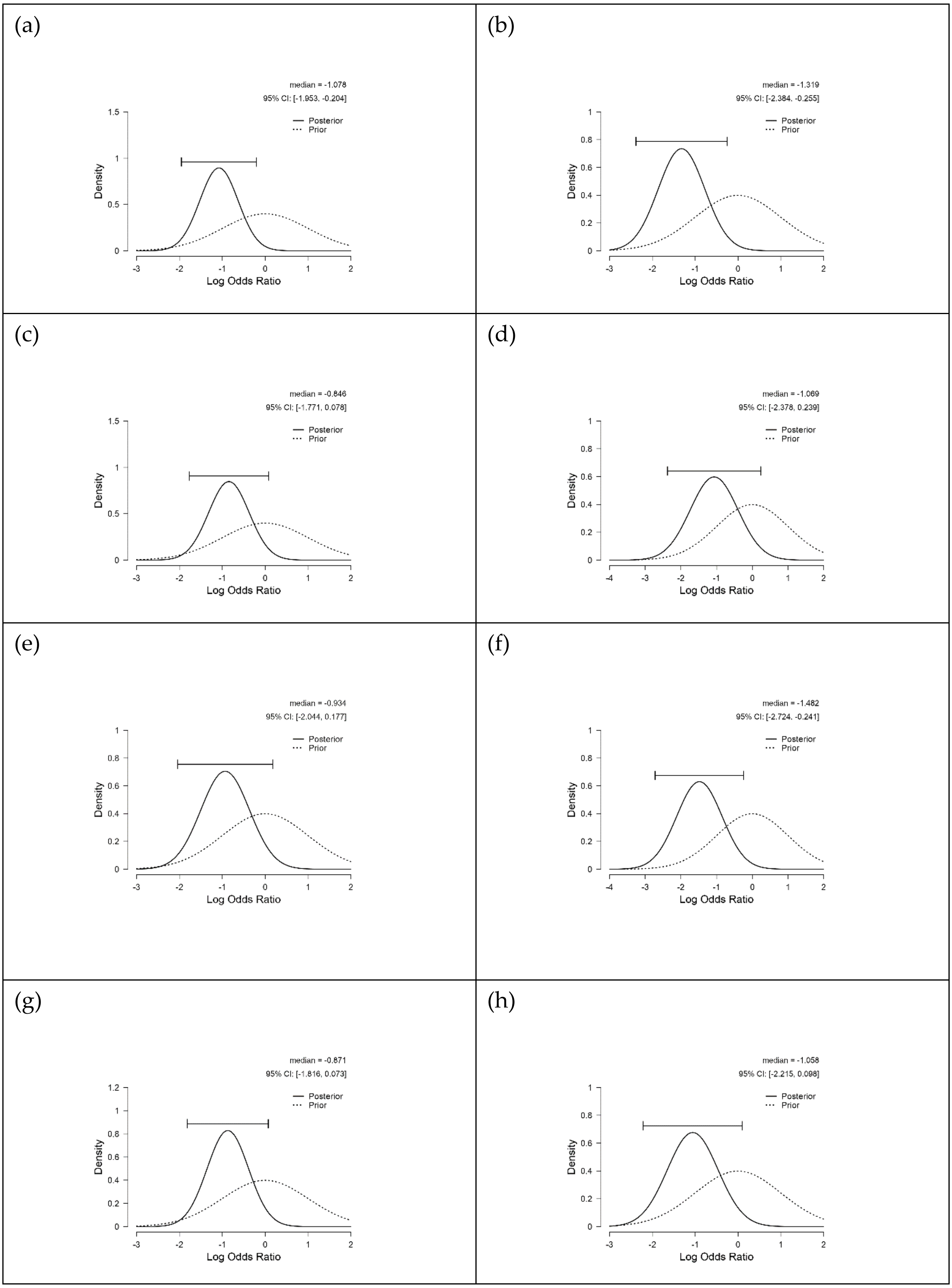

(a) CHK2 expression and response to therapy; (b) CHK2 expression and final follow-up; (c) CHK2 expression and tumor diameter; (d) CHK2 expression and PTC histologic variant; (e) *TP53* gene status and age; (f) *TP53* gene status and vascular invasion; (g) p53

expression + *TP53* gene status and age; (h) p53 expression and *TP53* gene status and vascular invasion. Abbreviation: 95% CI, 95% credible interval.

Figure S2: Sequential analysis for the relationships between clinicopathological features and CHK2 expression, *TP53* gene status, and p53 expression together with *TP53* gene status in the tumor tissues of PTC patients with and without *CHEK2* germline mutations

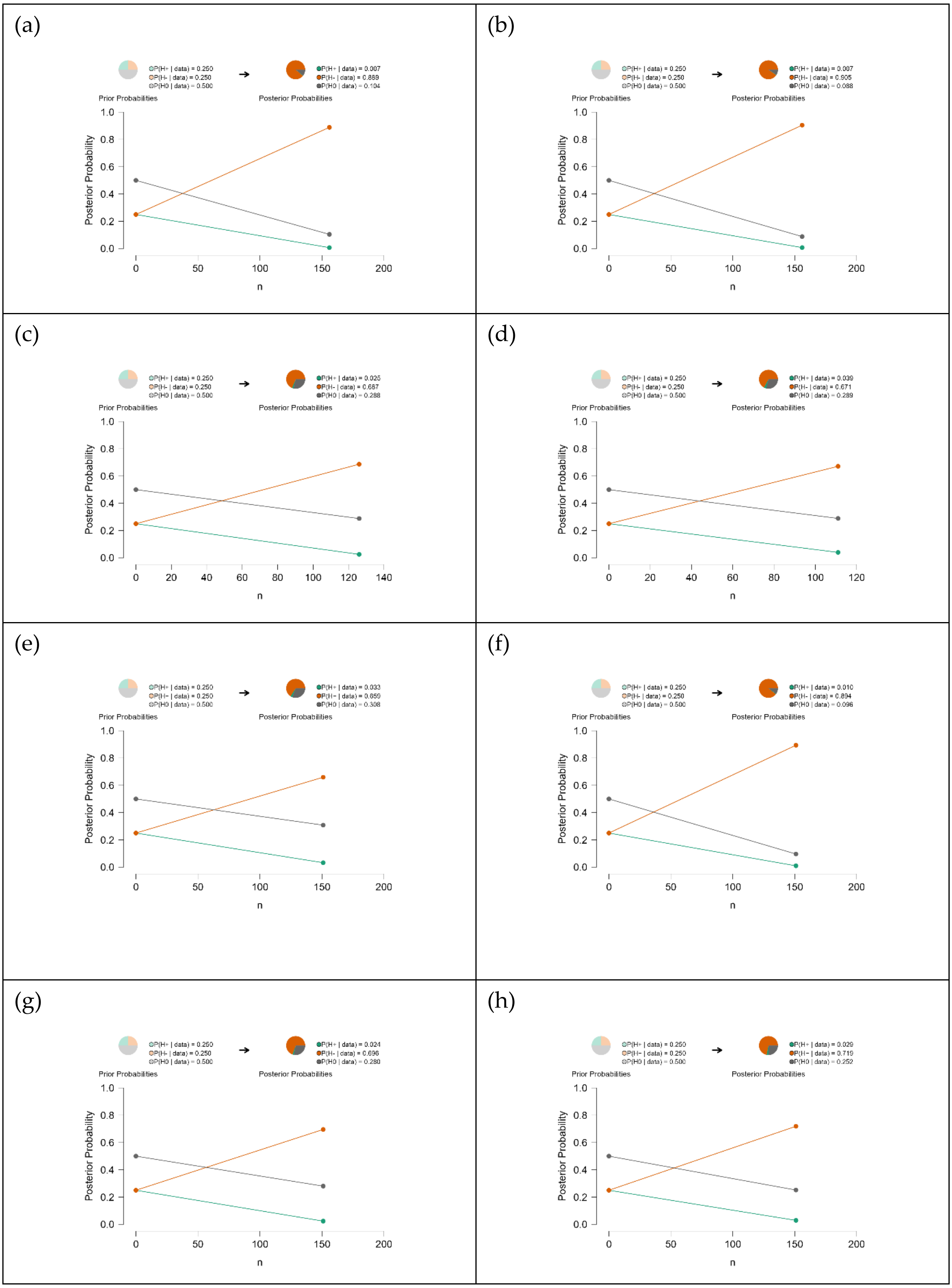

(a) CHK2 expression and response to therapy; (b) CHK2 expression and final follow-up; (c) CHK2 expression and tumor diameter; (d) CHK2 expression and PTC histologic variant; (e) *TP53* gene status and age; (f) *TP53* gene status and vascular invasion; (g) p53 expression together with *TP53* gene status and age; (h) p53 expression together with *TP53* gene status and vascular invasion. Abbreviations: P(H+|data), the probability of hypothesis H+ given the data; P(H-|data), the probability of hypothesis H- given the data; P(H0|data), the probability of hypothesis H0 given the data.

Figure S3: Posterior distribution of the odds log ratio for the association between *CHEK2* gene status, CHK2 expression, and the p53 expression together with *TP53* gene status in tumor tissues from PTC patients with and without the *CHEK2* germline mutations

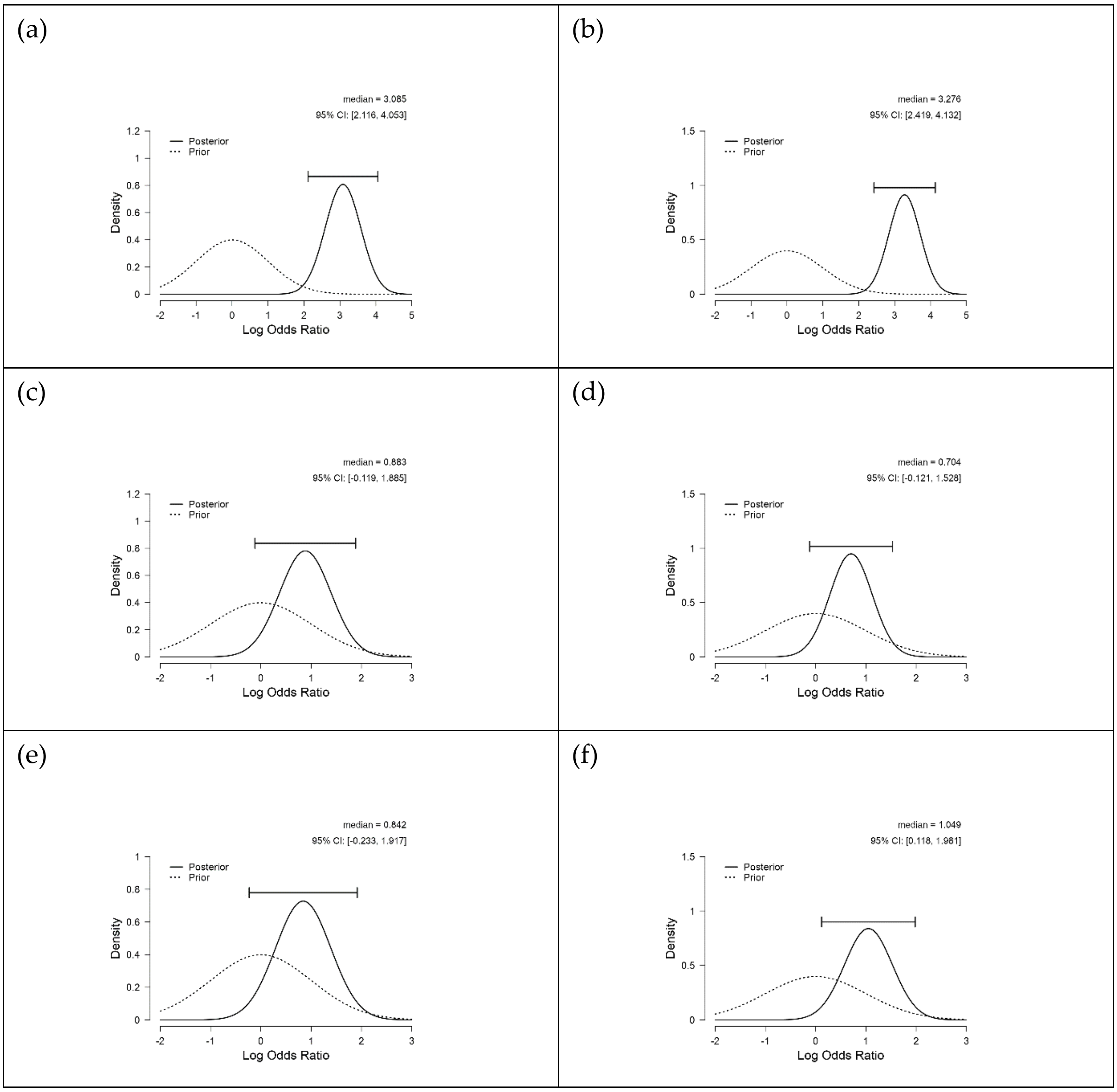

(a) *CHEK2* gene status vs. *CHEK2* truncating mutation and WT; (b) *CHEK2* gene status vs. *CHEK2* truncating mutation and WT group + missense I157T mutation group; (c) *CHEK2* expression vs. *CHEK2* truncating mutation and WT; (d) *CHEK2* expression vs. *CHEK2* truncating and WT group + missense I157T mutation group; (e) p53 expression and *TP53* gene status vs. *CHEK2* truncating mutation and WT; (f) p53 expression and *TP53* gene status vs. *CHEK2* expression vs. *CHEK2* truncating mutation and WT group + missense I157T mutation group Abbreviations: 95% CI, 95% credible interval; *CHEK2* gene status, no loss/deletion of a *CHEK2* gene copy; *TP53* gene status, no loss/deletion of a *TP53* gene copy as determined by FISH; FISH, fluorescence in situ hybridization; p53 expression, negative/positive as determined by IHC; IHC, immunohistochemistry; *CHEK2* truncating, germline heterozygous truncating *CHEK2* mutation variants (1100delC, IVS2+1G>A, del5395); WT, wild type.

Figure S4: Sequential analysis of the associations between *CHEK2* gene status, CHK2 expression as determined by IHC, and p53 expression together with *TP53* gene status as determined by FISH in tumor tissues from PTC patients with and without the *CHEK2* germline mutations

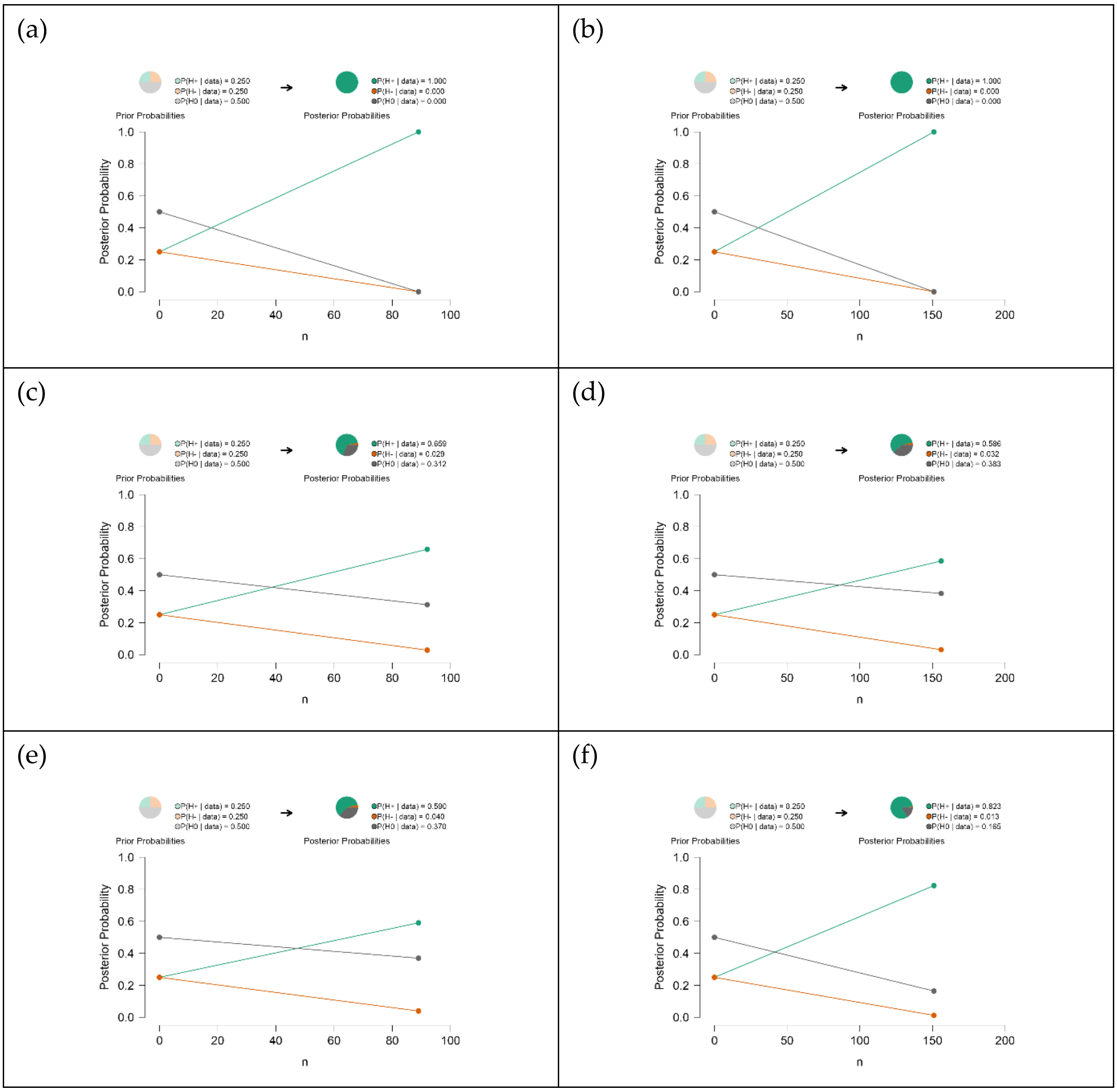

(a) *CHEK2* gene status vs. *CHEK2* truncating mutation and WT; (b) *CHEK2* gene status vs. *CHEK2* truncating mutation and WT group + missense I157T mutation group ; (c) *CHEK2* expression vs. *CHEK2* truncating mutation and WT; (d) *CHEK2* expression vs. *CHEK2* truncating mutation and WT group + missense I157T mutation group; (e) p53 expression and *TP53* gene status vs. *CHEK2* truncating mutation and WT; (f) p53 expression and *TP53* gene status vs. *CHEK2* expression vs. *CHEK2* truncating mutation and WT group + missense I157T mutation group.Abbreviations: 95% CI, 95% credible interval; *CHEK2* gene status, no loss/deletion of a *CHEK2* gene copy; *TP53* gene status, no loss/deletion of a *TP53* gene copy; FISH, fluorescence in situ hybridization; p53 expression, negative/positive as determined by IHC; IHC, immunohistochemistry; *CHEK2* truncating, germline heterozygous truncating *CHEK2* mutation variants (1100delC, IVS2+1G>A, del5395); WT, wild type; P(H+|data), the probability of hypothesis H+ given the data; P(H-|data), the probability of hypothesis H- given the data; P(H0|data), the probability of hypothesis H0 given the data.

Figure S5: CHK2 expression levels in primary papillary thyroid cancer tissue and lymph node metastases.

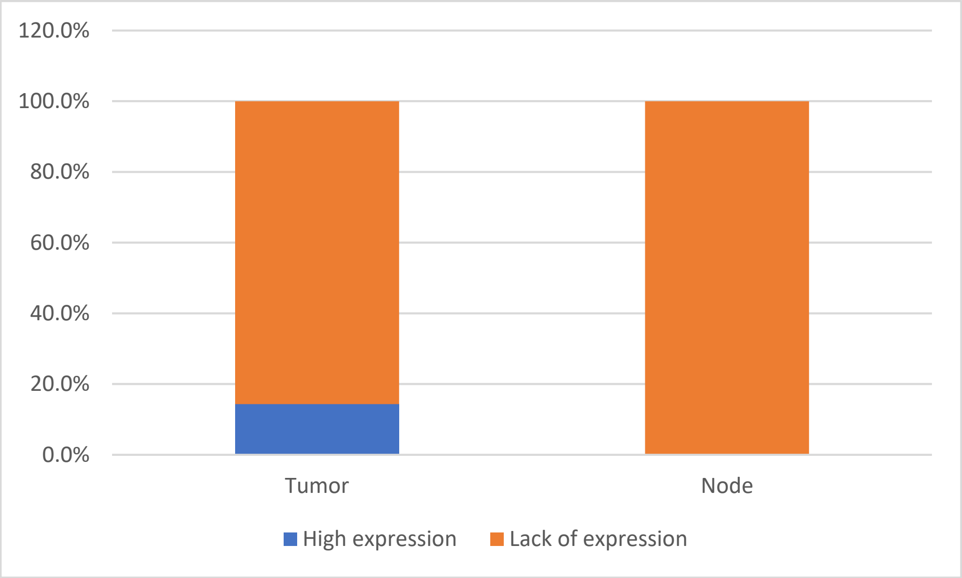

Table S1: Predictive values of the tested hypotheses for the relationships between clinicopathological features, treatment response, and disease outcome and level of CHK2 expression and *TP53* gene status, and p53 expression together with *TP53* status in tumor tissues from PTC patients with and without *CHEK2* germline mutations.

Variables	Models	P(M)	P(M∣data)	BF_{10}
CHK2 expression and response to therapy	Log odds ratio = 0	0.500	0.104	1.000
	Log odds ratio > 0	0.250	0.007	0.135
	Log odds ratio < 0	0.250	0.889	17.031
CHK2 expression and final follow-up	Log odds ratio = 0	0.500	0.088	1.000
	Log odds ratio > 0	0.250	0.007	0.168
	Log odds ratio < 0	0.250	0.905	20.554
CHK2 expression and tumor diameter	Log odds ratio = 0	0.500	0.288	1.000
	Log odds ratio > 0	0.250	0.025	0.174
	Log odds ratio < 0	0.250	0.687	4.766
CHK2 expression and PTC histologic variant	Log odds ratio = 0	0.500	0.289	1.000
	Log odds ratio > 0	0.250	0.039	0.273
	Log odds ratio < 0	0.250	0.671	4.646
TP53 gene status and age	Log odds ratio = 0	0.500	0.308	1.000
	Log odds ratio > 0	0.250	0.033	0.212
	Log odds ratio < 0	0.250	0.659	4.280
TP53 gene status and vascular invasion	Log odds ratio = 0	0.500	0.096	1.000
	Log odds ratio > 0	0.250	0.010	0.208
	Log odds ratio < 0	0.250	0.894	18.553
p53 expression + *TP53* gene status and age	Log odds ratio = 0	0.500	0.280	1.000
	Log odds ratio > 0	0.250	0.024	0.170
	Log odds ratio < 0	0.250	0.696	4.962
p53 expression + *TP53* gene status and vascular invasion	Log odds ratio = 0	0.500	0.252	1.000
	Log odds ratio > 0	0.250	0.029	0.233
	Log odds ratio < 0	0.250	0.719	5.713

Abbreviations: P(M), prior model probability; P(M|data), posterior model probability; BF_{10}, Bayes factor giving the evidence for H1 over H0; CHK2 expression, CHK2 expression (loss/low/high) as determined by IHC; *TP53* gene status, *TP53* no loss/deletion of a gene copy in tumor tissue as determined by FISH; FISH, fluorescence in situ hybridization; p53, positive/negative p53 expression as determined by IHC; IHC, immunohistochemistry

Table S2: Predictive value of tested hypotheses for the association between *CHEK2* gene status, CHK2 expression, and p53 expression together with *TP53* gene status in PTC patients with and without the *CHEK2* germline mutations

Variable		*CHEK2* truncating and WT			*CHEK2* truncating and WT + missense I157T		
	Models	P(M)	P(M|data)	BF_{10}	P(M)	P(M|data)	BF_{10}
CHEK2 gene status	Log odds ratio = 0	0.500	1.123e-10	1.000	0.500	1.898e-15	1.000
	Log odds ratio > 0	0.250	1.000	1.781e+10	0.250	1.000	1.054e+15
	Log odds ratio < 0	0.250	2.610e-12	0.046	0.250	3.282e-17	0.035
CHK2 expression	Log odds ratio = 0	0.500	0.312	1.000	0.500	0.383	1.000
	Log odds ratio > 0	0.250	0.659	4.215	0.250	0.586	3.058
	Log odds ratio < 0	0.250	0.029	0.185	0.250	0.032	0.165
p53 expression and *TP53* gene status	Log odds ratio = 0	0.500	0.370	1.000	0.500	0.165	1.000
	Log odds ratio > 0	0.250	0.590	3.194	0.250	0.823	9.999
	Log odds ratio < 0	0.250	0.040	0.215	0.250	0.013	0.152

Abbreviations: *CHEK2* gene status, no loss/deletion of a *CHEK2* gene copy; *TP53* gene status, no loss/deletion of a *TP53* gene copy as determined by FISH; FISH, fluorescence in situ hybridization; p53 expression, negative/positive as determined by IHC; IHC, immunohistochemistry; *CHEK2* truncating, germline heterozygous truncating *CHEK2* mutation variants (1100delC, IVS2+1G>A, del5395); WT, wild type.
